# Machine learning approaches for elastic localization linkages in high-contrast composite materials

**DOI:** 10.1186/s40192-015-0042-z

**Published:** 2015-12-04

**Authors:** Ruoqian Liu, Yuksel C. Yabansu, Ankit Agrawal, Surya R. Kalidindi, Alok N. Choudhary

**Affiliations:** 1 Department of Electrical Engineering and Computer Science, Northwestern University, Evanston, 60208 IL USA; 2 George W. Woodruff School of Mechanical Engineering, Georgia Institute of Technology, Atlanta, 30332 GA USA; 3 College of Computing, Georgia Institute of Technology, Atlanta, 30332 GA USA

**Keywords:** Materials informatics, Data mining, Elastic localization linkages, Structure feature selection, Structure feature ranking, Ensemble-based regression

## Abstract

There has been a growing recognition of the opportunities afforded by advanced data science and informatics approaches in addressing the computational demands of modeling and simulation of multiscale materials science phenomena. More specifically, the mining of microstructure–property relationships by various methods in machine learning and data mining opens exciting new opportunities that can potentially result in a fast and efficient material design. This work explores and presents multiple viable approaches for computationally efficient predictions of the microscale elastic strain fields in a three-dimensional (3-D) voxel-based microstructure volume element (MVE). Advanced concepts in machine learning and data mining, including feature extraction, feature ranking and selection, and regression modeling, are explored as data experiments. Improvements are demonstrated in a gradually escalated fashion achieved by (1) feature descriptors introduced to represent voxel neighborhood characteristics, (2) a reduced set of descriptors with top importance, and (3) an ensemble-based regression technique.

## Background

Material data sciences and informatics [1–12] are emerging as foundational disciplines in the realization of the vision set forth in various high-profile national strategic documents [13, 14]. The novel tools developed in these emerging fields focus mainly on transforming large amounts of collected data (from both experiments and computer simulations) into higher value knowledge that can also be easily disseminated to the broader research community. More specifically, various emerging concepts and tools in machine learning and data mining methods are applied to represent, parse, store, manage, and analyze material data. The higher value knowledge extracted using these tools can be used to dramatically accelerate material development efforts for a range of advanced technologies. One of the central tasks in the analyses of materials data is the identification and extraction of robust and reliable structure–property relationships [15–33].

The internal structure of a material system exhibits multiple hierarchical length scales that play a pivotal role in the behavior and performance characteristics of the material. Consequently, multiscale modeling is an integral component of any effort aimed at rational material design. Almost all multiscale models currently employed in materials design involve one-way coupling, where the information is passed mainly from a lower to a higher length scale (also called homogenization). Communication of high-value information in the opposite direction (also called localization) is usually very limited. For the purpose of achieving efficient scale bridging, data-driven approaches for establishing localization structure–property relationships as low-computational-cost linkages (i.e., surrogate models or metamodels) are of great interest.

Physics-based multiscale material models provide tools needed to explore the role of material structure in optimizing the overall (effective) properties of interest. This is generally accomplished by solving governing field equations numerically (e.g., finite element models), while satisfying the appropriate (lower length scale) material constitutive laws and the imposed boundary and initial conditions. However, the computational resource requirements of such multiscale materials models are usually very high, rendering these tools impractical for the needs of rational material design and optimization. Besides the high computational requirements, there is not enough attention paid to systematic learning from these simulations. In other words, in any typical design and optimization effort, solutions of the governing field equations are generally obtained for multiple trials of the material structures. However, most solutions that do not produce the desired property or performance are routinely discarded without distilling transferable knowledge from them. It is extremely important to recognize that even when the trial did not produce the desired solution, there is a great deal of information in the solution obtained. Since a significant computational cost was expended in arriving at the solution, it only behooves us to learn as much as we can from the solution obtained. Machine learning techniques and data-driven methods are ideally suited for this task and can lead to dramatic savings in both time and effort, when implemented properly into the material development efforts. In the present study, we demonstrate the implementation of one such strategy for capturing the elastic localization in high-contrast composite material systems in a low-cost surrogate model that is applicable to a very broad set of potential material internal structures.

Materials informatics is an emerging discipline that leverages information technology and data science to uncover the essential process–structure–property (PSP) relationships central to accelerated discovery and design of new/improved materials. A large part of materials informatics involves the use of data mining and machine learning techniques to exploit materials databases and discover trends and mathematical relations for material design [34]. Data-centered methods, as opposed to ab initio methods, are generally expressed as heuristic models, statistically learned from large amounts of historically accumulated observations. Bearing sound generality, they are also able to adapt quickly to new observations. The capability of establishing models from a pure statistical or “machine-like” standpoint avoids human interference and thus enhances the chance of finding the embedded high-value information in an objective manner, especially when this knowledge is not easily expressed through simple equations.

The rich complexity of the material internal structure typically demands a high-dimensional representation [3, 5, 10, 20, 35, 36]. In general, it is actually preferable to start with a more than sufficient list of potential descriptive features (interpreted here as measures of material internal structure) prior to building the models. In this phase of model building, it is fully acknowledged that the salient features are only expected to naturally lie in a much lower dimensional space. An important step of machine learning is the identification of these salient features using suitable feature selection techniques or a transformation of features from a higher dimensional space to a lower dimensional space, known as feature extraction. Both selection and extraction can be either supervised or unsupervised. If the response of the material structure (e.g., the elastic response associated with the material structure in the present case study) needs to be predicted, supervised learning provides more insights in the selection process.

Our interest in the present paper is in building data-centered localization linkages to predict elastic deformation fields in a high-contrast two-phase composite system. For the present study, contrast refers to the ratio of the elastic stiffness parameters of the constituent phases of the composite system. For isotropic constituents, contrast usually refers to the ratio of the Young’s moduli of the phases present in the composite material. As the contrast decreases, the interactions between the microscale constituents become less severe and therefore less significant. Thus, the errors are expected to be considerably lower with lower contrasts. The error measures and results for lower contrast materials systems have been reported in prior work [15, 19, 22, 33]. More specifically, our goal here is to mine localization linkages from an accumulated set of observations and then use the extracted models to predict the response in new, not yet analyzed, structures. In this pursuit, we will explore the use and adaptation of machine learning systems specifically tailored to large-scale datasets and high-dimensional problems. More specifically, three key data experiments are designed and conducted in progression leading finally to highly robust localization linkages for the high-contrast composites studied:

- Features identifying the local neighborhood of a voxel to different degrees of adequacy are explored systematically with carefully defined neighbor levels.
- Multiple strategies are explored for ranking the large number of potential features that could be used to quantify the neighborhood of the voxel of interest.
- Different strategies for formulating regression models are critically evaluated and contrasted for their computational efficacy and accuracy for the selected task. Ensemble methods, which aggregate a number of weak regressors each specializing in a subdomain of the original task, have shown substantial promise.

## Methods

### Problem statement

Localization, as opposed to homogenization, describes the spatial distribution of the response at the microscale for an imposed loading condition (e.g., averaged strain) at macroscale. Localization is critically important in correlating various failure-related macroscale properties of the material with the specific local microstructure conformations responsible for the (local) damage initiation in the material. In this work, these two scales are to be connected through linkages extracted by data-driven processes used in machine learning systems.

More specifically, we focus our effort in this study on extracting localization relationship for elastic deformation in a two-phase composite [15, 16, 18, 19]. The input into such a linkage typically includes the material microstructure (defined in a three-dimensional (3-D) microscale volume element (MVE)) and the applied macroscale loading condition (typically expressed as the averaged elastic strain imposed on the MVE). The output from the linkage is the microscale elastic strain field throughout the MVE.

The first step in the application of data science methods is the collection and organization of appropriate data from which the linkages can be mined efficiently. At the present time, suitable datasets for this purpose can only be obtained using numerical models. The experimental protocols for measuring 3-D stress (or strain fields) are still very much in developmental stages [37–39]. Therefore, we proceed here with datasets created by numerical physics-based models (e.g., finite element (FE) models). In other words, we consider the predictions obtained by the FE models as the “ground truth” and we want to establish the localization linkages as a surrogate model for the actual FE model. Our expectation is that the surrogate model will provide a much faster answer compared to the FE model with only a modest loss in accuracy.

**Fig. 1:**
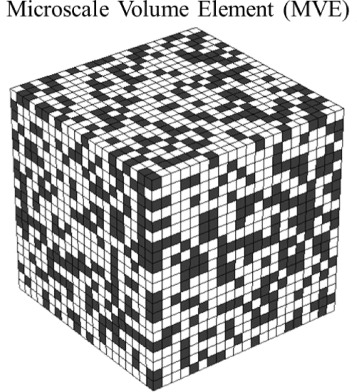
An example microscale volume element (MVE). Each voxel in the MVE is fully occupied with either black or white phase

In this work, we first produced a dataset containing a large ensemble of digitally created 3-D microstructures. Each 3-D microstructure is defined on a uniformly tessellated spatial grid and is referred as a microstructure volume element (MVE). Each MVE is transformed into a FE model, where each spatial cell (i.e., voxel) is converted to an element of the FE mesh. The response of each MVE was then computed employing standard protocols based on the use of periodic boundary conditions and the commercial finite element software, ABAQUS [40]. Periodic boundary conditions were set in all six faces of the MVEs for all three displacements. In this study, the strain component of interest was selected as *ε*_*xx*_. Hence, the periodic boundary conditions were applied to MVEs in such a way that only the applied macroscopic strain for this component was nonzero; this was done by setting a difference in the *x* component of the displacement only on the faces perpendicular to the *x* direction. With these conditions, all other strain components at macroscopic level become zero. The same approach we used for *ε*_*xx*_ strain component can be repeated for all six strain components for a full set of linkages that would serve for any arbitrary loading condition while exploiting the superposition principle. Further details regarding these periodic boundary conditions and the approaches described above can be found in our prior work [15]. For the present study, following protocols used in prior studies, each MVE was selected to consist of 21×21×21=9261 voxels [15, 22, 33]. Each element in the MVE is assigned one of the two possible phases depicted as black and white in Fig. 1 (associated with values 0 and 1, respectively, in the description of the microstructure), while the response field is captured as a continuous number on the same spatial grid (one average value for each element of the FE model) that was used to define the microstructure or the MVE. Both constituent phases of the composite are assumed to exhibit isotropic elastic response with Young’s modulus, *E* = 12 GPa and *ν*= 0.3 for the black phase and *E* = 120 GPa and Poisson ratio, *ν*= 0.3 for the white phase. Note that this assignment of properties for the individual phases of the composite system corresponds to a contrast ratio of 10 (this is the ratio of the Young’s moduli of the two phases present in the composite). It should be noted that most of the prior work in this area has largely focused on composites with significantly lower contrast ratios of about 2 [15]. There has only been one previous work reported in the literature thus far with a contrast ratio of 10 [18]. However, in that prior study, the feature selection was addressed using heuristics, significantly different from the data science approaches presented in this work.

### Design of data experiments

**Fig. 2:**
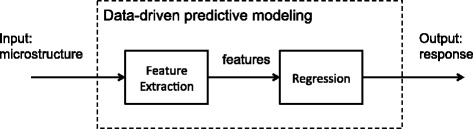
A simplified data-driven modeling flowchart. It is comprised of two steps, feature extraction, and regression, both studied in later data experiments

Figure 2 schematically illustrates the main data-driven protocol for establishing a predictive model. It generally comprises of two key processes: (i) feature extraction, and (ii) construction of the regression model. Each process requires numerous trials that are generally referred to as data experiments. In this work, we have conducted two data experiments for the feature extraction process and a third data experiment for the construction of the regression model. The design of these three data experiments are detailed later in this section.

A total of 2500 MVEs with varying volume fractions were included in this study. They are evenly distributed in 100 variations of volume fraction values, from 1.0 to 99.4 %. Therefore, 25 MVEs are present in each variation, within which, 15 are used as calibration (for feature extraction, model training), and the remaining 10 are used for validation.

The data experiments were carried out on a Linux Red Hat 4.4.7 system with 32-GB memory and Intel Xeon CPU 2.20 GHz. A Python-based machine learning library, scikit-learn [41], is used in most implementations (except the M5 model tree is implemented in a C library). The performance of the models was evaluated by the mean absolute strain error (MASE) *e* in a MVE, defined as

(1) $$ e = \frac{1}{S} \sum_{s=1}^{S} \lvert \frac{p_{s}-\hat p_{s}}{p_{\text{imposed}}} \rvert \times 100~\%  $$

where *p*_imposed_ denotes the average strain imposed on the MVE, and *p*_*s*_ and $\hat p_{s}$ denote the values of the strain in the voxel *s* from the FE model and the surrogate model developed in this work, respectively. This metric quantifies the average error for a single MVE microstructure. In the data experiments presented here, we show both individual *e* for each MVE as well as averaged MASE, $\bar e$, over the entire set of 1000 validation MVEs.

In constructing training and test data for predictive modeling, each voxel in the MVE is examined, represented, and transformed into a data instance consisting of “inputs” and “outputs”. Each MVE generates 9261 data samples (this is the number of voxels in each MVE). The complete calibration set hence contains 13,891,500 samples and validation contains 9,261,000 samples.

We term the voxel under examination as the “focal voxel”, whose response (average elastic strain in the voxel) is to be predicted. Each voxel in the MVE gets to be the focal voxel once, and when it does, other voxels in its local environment are taken to construct input features for it. By doing this, we are assuming that the response of a focal voxel is strongly influenced by some short-range interactions with neighboring voxels in its local environment. This concept is highly consistent with the concepts of Green’s functions utilized extensively in composite theories [42–47].

Following the symbolic definitions in [15–19, 22], we let the microstructure variables ${m_{s}^{0}}$ and ${m_{s}^{1}}$ denote the volume fraction of each local state in each voxel of the composite MVE, where 0<*s*≤*S* indexes the voxels; *S*=9261 is the total number of voxels in an MVE. Since ${m_{s}^{1}}+{m_{s}^{0}} = 1$ and we employ eigenmicrostructures (each voxel is assigned exclusively to one of the two phases allowed) in the present case study, we further simplify the notation and use *m*_*s*_ to simply denote ${m_{s}^{1}}$ in some of the case studies presented here.

As noted earlier, the averaged local response (elastic strain) in each voxel in presented as *p*_*s*_, where 0<*s*≤*S*, *S* being the total number of voxels in the MVE. We expect that not only the value of *p*_*s*_ is influenced by *m*_*s*_ but also the value of the microstructure function in the voxels in the neighborhood of *s*. We use the notation *m*_*s,l,t*_ to refer to the microstructure function values in the neighborhood of *m*_*s*_, where *l* refers to the neighbor level (defined based on distance from *s*) and *t* refers to individual voxels in the layer *l*. These concepts are further elaborated below.

**Fig. 3:**
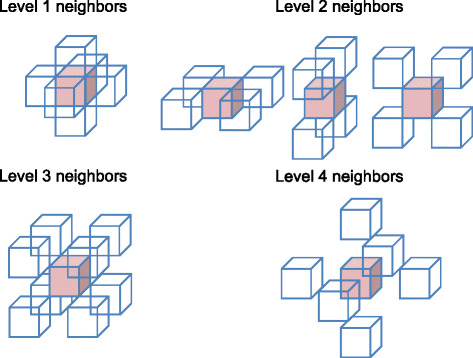
Illustration of the neighbor positions at 1–4 levels
- Individual voxel *t* in a neighbor layer. In each layer of the same neighbor level, there can be none or a number *T*_*l*_ of neighbor member voxels. As shown in Fig. 3, there are *T*_1_=6 first-level neighbors, *T*_2_=12 second-level neighbors, *T*_3_=8 third-level neighbors, and *T*_4_=6 fourth-level neighbors. To address each of them, we assign an index variable *t*=0,…,*T*_*l*_−1. For example, all voxels at neighbor level 1 of *s* can be indexed as (*s*,1,0), (*s*,1,1), (*s*,1,2), (*s*,1,3),(*s*,1,4), and (*s*,1,5), following the notation introduced earlier.

- Level of neighbors, *l*. Neighbors generally refer to voxels adjoining a given voxel. Here, we extend the definition and serialize neighbors based on their scalar distances from the voxel of interest. Figure 3 shows a 3-D voxel of interest in pink, surrounded by its different levels of neighbor voxels. The level of a neighbor, *l*, is used in this study to identify all of the voxels that are at a distance of $\sqrt {l}$ from the voxel of interest. In Fig. 3, *l*=1, 2, 3, 4 from the upper left to the lower right. In this work, where MVEs are of dimension 21×21×21, a voxel can have up to 300 levels of neighbors, although, at some of these levels, there do not exist any neighbor members (for example, *l*=7 and *l*=15 do not have any members invalid as their squared values cannot be represented by a sum of squares of 3 whole numbers).

Following this nomenclature, *m*_*s*,0,0_ is the (binary) microstructure variable at *s*, i.e., the focal voxel. Its neighboring voxels, *m*_*s,l,t*_, along with other extracted feature variables are included in the input feature vector when modeling *p*_*s*_.

Three data exercises are designed and conducted here to study the important subprocesses involved in building a data-centered learning system for localization: (i) neighbor inclusion—how large a spatial neighborhood of voxels should be considered in formulating the statistical model for the response at the focal voxel; (ii) feature extraction—what salient features should be considered in building simplified geometrical constructs among the neighborhood voxels; and (iii) regressors—what learning algorithm should be used for connecting the microstructure and the desired local response.

#### Design of exercise 1

In this first exercise, namely, Ex 1, we focus on identifying the amount of information needed in forming an accurate representation of a focal voxel, with its local neighborhood. By only using the structure information given by *m*_*s,l,t*_, we explore how much of a *l* is necessary in order to represent adequately the neighborhood of *m*_*s*,0,0_ for the elastic localization linkages of interest. As we increase *l*, the number of input variables used in the modeling *p*_*s*_ will also increase.

Six variations are designed, varying the number of inputs by adjusting the extent to which level neighbors are to be included. Only input features are varied, and the prediction target (*p*_*s*_) and regression scheme are fixed. A M5 model tree, which is a type of decision tree with linear regression functions at the leaves, is used as the regression model for the data experiments in this case study. The M5 model tree is based on the M5 scheme described by Quinlan [48] and implemented by Wang and Witten [49]. This set of experiments is aimed at answering the question: *Will using more information about the neighbors’ help improve the prediction model for the elastic response at the focal voxel?*

#### Design of exercise 2

In this exercise, we explore the design and identification of features that provide a more complete representation of the microstructure. The full list of potential features designed is shown in Table 1 and is further explained below. The purpose of these constructed features is to account for not only the individual values of *m*_*s,l,t*_ in the neighborhood of the focal voxel but also certain aggregated neighborhood features that might be more efficient in capturing the desired linkages. Examples of such constructed features may include the distribution of ${m_{s}^{1}}$ and ${m_{s}^{0}}$ at (or up to) each neighbor level *l* and the symmetry of a local structure, among several others. The following specific ones (see also Table 1) have been explored in this exercise:

- *m*_*s,l,t*_ is what has been used in Ex 1, the microstructure value of voxels in the neighborhood of *s*. We use up to the 12th level, and the total number of neighbor voxels are 1+6+12+8+…=179.

**Table 1:** Definition of the set of features constructed in Ex 2, with regard to the representation of a focal voxel at *s*

Symbol	Meaning	Count	Scope
*m*_*s,l,t*_	Microstructure value of voxels at a neighbor level *l*, with index *t*, of a focal voxel at *s*	179	Binary, {0,1}
	*l*=1,…,12		
$\text {pr}_{l}^{h}$	Fraction of voxels with microstructure phase *h* at neighbor level *l*	24	Real, [0,1]
$\text {pr}_{l}^{h}$	Fraction of voxels with microstructure phase *h* up to neighbor level *l*	24	Real, [0,1]
$I_{\text {norm}}^{h}$	The normalized impact of all 12 levels of neighbors of phase *h*	2	Real
	$ I_{\text {norm}}^{h} = \sum _{i=1}^{12} T_{l}\cdot \text {pr}_{l}^{h}/\sqrt {l} + T_{0}\cdot \text {pr}_{0}^{h}/0.5$		
*S*_3_	3-plane symmetry index	1	Real
*S*_9_	9-plane symmetry index	1	Real
- $\text {pr}_{l}^{h}$ is the volume fraction of phase *h* in neighborhood level *l*.
- $\text {pr}_{l}^{h}$ is the accumulated volume fraction of phase *h* up to neighborhood level *l*.
- $I_{\text {norm}}^{h}$ is defined as the aggregated “impact” to a focal voxel of all its neighbors up to a specified level (in this exercise, we include up to the 12th level). For this purpose, we first quantify the impact of each voxel in neighbor level *l* to be given by $1/\sqrt {l}$; as expected, closer neighbors have higher impact values. For all voxels at *l* (*l*>0), the overall impact is computed as ${I_{l}^{h}}=T_{l}\cdot \text {pr}_{l}^{h}/\sqrt {l}$. For *l*=0, the impact value is assigned as ${I_{0}^{h}}=2$. $I_{\text {norm}}^{h}$ is then calculated as a sum of impacts from all levels (up to 12), $I_{\textit {norm}}^{h}=\sum _{i=0}^{12}{I_{i}^{h}}$. It is easy to see that the sum of $I_{\text {norm}}^{0}$ and $I_{\text {norm}}^{1}$ is always a constant value ($=2.0+T_{1}/\sqrt {1}+T_{2}/\sqrt {2}+T_{3}/\sqrt {3}+\dots $) where *T*_1_=6,*T*_2_=12,*T*_3_=8,….
- *S*_3_ and *S*_9_ stand for two symmetry descriptors looking at a 3-D local microstructure, including up to the 12 neighbor levels, centered at the focal voxel. Symmetry is defined as the degree of similarity between the two halves of the 3-D structure when bisected by a specified plane. *S*_3_ considers three dividing planes passing through the center focal voxel, and *S*_9_ uses nine, adding six diagonal ones. Planes are illustrated in Fig. 4, where the focal voxel is placed at the center of the structure. Note that the MVE structure in the figure is only for illustration. In actual calculation, planes cut through an irregular but symmetrical structure where a focal voxel is in the center and all of its neighbors up to the 12th level (in total, 178 neighbor voxels) scatter around it. For every dividing plane, we assess how similar the resulting two half-structures are to each other, by computing a voxel-to-voxel exclusive nor (XNOR, giving one when two voxels are the same) of the two half-structures and then taking a distance-normalized sum. In this way, nonconformity farther away from the focal voxel has a smaller effect.

**Fig. 4:**
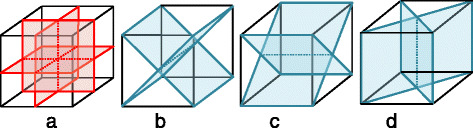
Planes used to define symmetry measures *S*_3_ and *S*_9_. The three red planes in **a** are used to obtain *S*_3_, whereas for *S*_9_, all nine planes in **a**, **b**, **c**, and **d** are used

**Fig. 5:**
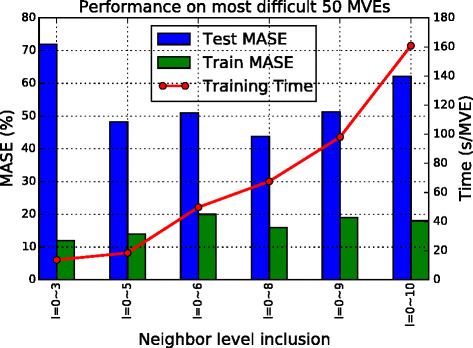
Results of Ex 1. Comparison of training time (*line*) and training and test errors (*bars*) with different numbers of microstructure inputs. Six variations are tested. Results shown are only for the 50 MVEs with volume fraction 48–53 % (these produce the highest errors). Most systems turn out to have a test error $\bar {e}$ of over 50 %, which indicates the insufficiency of representation

The entire set containing 231 feature variables are examined systematically for their effect on feature reduction. This is important because Ex 1 (see Fig. 5) demonstrated that including more features than needed can actually deteriorate the performance of the predictive model.

To produce a ranking of feature importance, we applied a filter method that employs Pearson’s correlation as a heuristic measure of feature quality. When a feature is continuous (all features except ${m_{l}^{h}}$), the standard Pearson’s correlation is applied:

(2) $$ r_{XY} = \frac{\sum (x-\mu_{x})(y-\mu_{y})}{k\sigma_{X}\sigma_{Y}},  $$

where *X* is the feature variable to be evaluated and *Y* is the target variable (i.e., the elastic strain at the focal voxel). In the above equation, *k* is the variable length, *μ* denotes the mean, and *σ* is the standard deviation. In the case of evaluating discrete features such as *m*_*s,l,t*_, the modified form, weighted Pearson’s correlation, is used:

(3) $$ r'_{XY} = P(X=0)r_{X_{0}Y}+P(X=1)r_{X_{1}Y},  $$

where *P*(*X*=*h*) is the prior probability that the microstructure *X* takes value *h* and *X*_*h*_ is a binary attribute that takes the value 1 when *X*=*h* and 0 otherwise.

We consider the correlation between a feature *X* and the prediction target *Y* as an indication of the relevance of *X* in building a predictive system for *Y*. By obtaining correlation coefficients for each *X*, a ranking is produced, seen in Table 2, where from top down, features with the best relevance quality are listed (top 30 are shown). Ex 2, comprised of Ex 2a, Ex 2b, Ex 2c, and Ex 2d, takes various numbers of top-ranked features in constructing prediction models.

**Table 2:** Features ranked by the correlation with the response. Top 30 are shown

Rank	Feature
1	*m*_*s*,0,0_
2–7	*m*_*s*,1,2_, *m*_*s*,1,3_, *m*_*s*,1,1_, *m*_*s*,1,0_, *m*_*s*,1,4_, *m*_*s*,1,5_
8–13	*m*_*s*,2,2_, *m*_*s*,2,3_, *m*_*s*,2,0_, *m*_*s*,2,1_, *m*_*s*,4,4_, *m*_*s*,2,4_
14–16	$\text {pr}_{1}^{1}$, *m*_*s*,2,4_, $\text {pr}_{1}^{0}$
17	$I_{\text {norm}}^{0}$
18	$\text {pr}_{1}^{1}$
19–20	*S*_9_, *S*_3_
21–23	*m*_*s*,2,8_, *m*_*s*,2,5_, *m*_*s*,3,3_
24	$\text {pr}_{1}^{0}$
25–30	*m*_*s*,2,6_, *m*_*s*,5,6_, *m*_*s*,5,10_, *m*_*s*,2,9_, *m*_*s*,8,28_, *m*_*s*,5,11_

#### Design of exercise 3

In the third exercise, we intend to investigate the effect of estimator models or learning algorithms in building a microstructure-response prediction system. Data experiments with various classical algorithms are designed. In addition to the M5 model tree, two more regressors are explored, identified as Ex 3a and Ex 3b and described below. The top 57 and 93 feature sets from Ex 2b and Ex 2c are used, as they provided the best models thus far. These two features sets are identified by appending −1 and −2, respectively, to the case studies. For example, Ex 3a −1 will utilize 57 feature inputs while Ex 3a −2 will utilize 93 feature inputs.

- *Ex 3a* As an extension to M5 regression tree, a random forest (RF) [50] regressor that forms an ensemble of many tree estimators is explored. The concept of ensemble learning or using a number of estimators and aggregating their results is expected to give a better generalization towards unseen data. The number of member estimators in RF is set to be 50.
- *Ex 3b* As a classic kernel-based learning model, support vector machine [51] finds an optimized hyperplane in feature space to separate classes. To deal with continuous class outputs, Support Vector Regression (SVR) [52] is used.

## Results and discussion

The following subsections present performances of each designed data experiment in terms of average prediction errors. Another measure of performance is the computational time. FEM simulations for each MVE took 23 s with two processors in a supercomputer, whereas with data models, once the model parameters are fixed, the prediction only takes a few milliseconds per MVE.

### Data exercise 1: neighbor inclusion

The first exercise studies the feature space constructed by neighbor voxels only. Since our goal at this point is to explore potential features for building the prediction model subsequently, it is not essential to use the entire dataset. In order to save computational cost, the six models (described earlier) are built and tested on a small subset that contained 50 MVEs with volume fractions of 48–53 %, which are regarded as the most difficult MVEs, because the response field exhibits the highest level of heterogeneity. Tenfold cross-validation is conducted where in each fold, 45 MVEs are used for training the model, and the remaining 5 MVEs are used for testing.

The results are summarized in Fig. 5, showing six variations in the inclusion of neighbor voxels *m*_*s,l,t*_ in building a relationship between *m*_*s*_ (or *m*_*s*,0,0_) and *p*_*s*_. *l* varies from 0 up to 3, 5, 6, 8, 9, and 10, from left to right in Fig. 5. This corresponds to a number of inputs of 27, 57, 81, 93, 123, and 147, respectively.

The results indicate that using more neighbors does not necessarily continue to enhance the accuracy. The model with an inclusion of neighbor level *l* up to 8 gives the best (least) test error. The speed of the learning of model trees is influenced linearly by feature dimensions.

Figure 5 also indicates that most of the experiments have a very high test error of over 50 %. The shortcoming of this series of modeling lies in the inadequacy of microstructure representation coming solely from individual components of the *m*_*s,l,t*_. In Ex 2, we aim to identify a set of engineered microstructure features in addition to *m*_*s,l,t*_ to represent more effectively the salient neighborhood features of the focal voxel.

### Data exercise 2: feature extraction

With the set (see Table 1) containing 231 feature variables devised, a series of exercises (labeled Ex 2) are conducted using different combinations of the feature variables based on a rank generated by correlation measures, while keeping the regression model the same. With regard to the rank of importance (partially shown in Table 2), we take various numbers of top features with the best relevance quality in constructing prediction models and thus designed Ex 2a, Ex 2b, Ex 2c, and Ex 2d. The top 27, 57, and 93 features are selected to match the number of inputs used in Ex 1, and the last exercise uses all 231 features in the set.

**Fig. 6:**
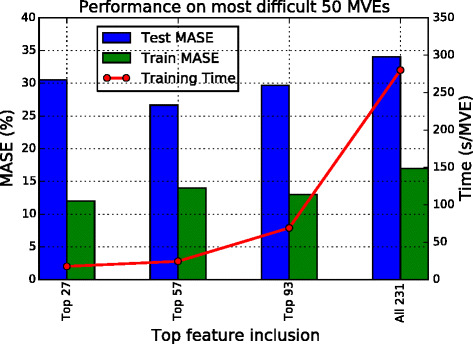
Results of Ex 2. Comparison of training time (*line*) and training and test errors (*bars*) with different numbers of top feature inclusions. The four systems from left to right correspond to Ex 2a, 2b, 2c, and 2d. Results shown are for 50 MVEs with volume fraction 48–53 %. Judging from the test MASE $\bar {e}$, the second system with 57 features outperforms the rest

To allow comparisons with Ex 1 (see Fig. 5), we take the same 50 MVEs to perform a tenfold cross-validation; the results obtained are shown in Fig. 6. Clearly, the models produced in Ex 2 are significantly better than those obtained in Ex 1.

**Fig. 7:**
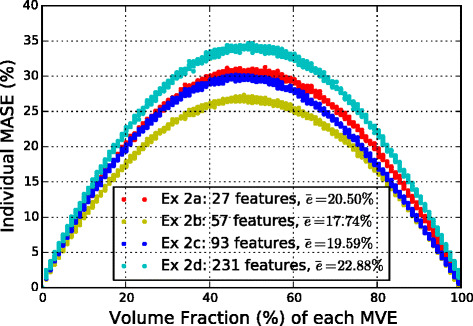
Influence of volume fraction on the error for Ex 2. Individual MASE for each of the 1000 MVEs in test set given by the four systems in Ex 2 are shown

Next, training is done on the entire training set of 1500 MVEs and tested on all 1000 test MVEs. Figure 7 shows the individual MASE for each of the 1000 MVEs from the test set, separated by the volume fraction. As expected, the model accuracy is highest at the low fractions (of either phases). Conversely, the highest error occurs in the volume fractions around 50 %, as the elastic strain fields in these composites are the most heterogeneous. Among the four sets of experiments, Ex 2b outperforms the rest both in terms of the average error rate and a reasonable training time.

**Fig. 8:**
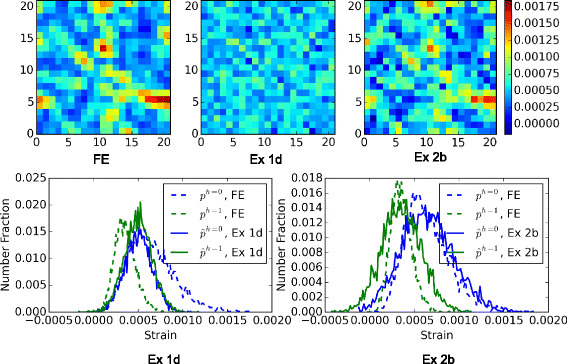
Comparison of strain field predictions. A comparison of FE and statistical model predictions of the strain fields, $\hat {p}$, in an example MVE with the volume fraction of 50 %. The models are those developed in Ex 1d and Ex 2b. *Strain fields* on a center slice are compared at the *top*. *Histogram plots* of strain distributions are presented at the *bottom*. One hundred bins are used to generate each distribution curve for each phase

A more direct comparison of the model results and the FE results are presented in Fig. 8. Only the model predictions from Ex 1d (this is the best of Ex 1) and Ex 2b (this is the best of Ex 2) are shown in this figure. In the top row of the figure are the elastic strain distributions in the middle slice of the MVE, and in the bottom are histogram plots of strain values predicted for the entire MVE. Two phases are separated in generating the distribution of the predicted strain values, each compared with FE distributions. One hundred bins are used, each of a width around 1e −05.

The example shown in this figure corresponded to a volume fraction of 50.22 % (this is one of the cases with the highest error). The improvements in the accuracy of Ex 2b over Ex 1d is clearly evident. In particular, it should be noted that Ex 2b is doing a very reasonable job in predicting the locations and distributions of the hot spots (voxels with the highest local elastic strain).

### Data exercise 3: regressors

**Fig. 9:**
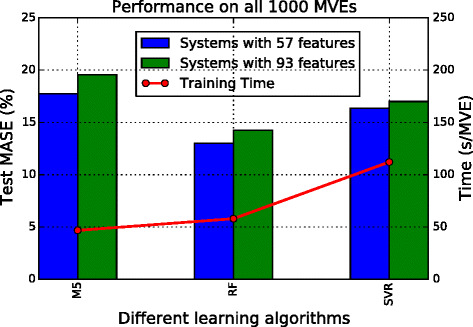
Results of Ex 3. Comparison of training time (*line*) and test error (*bars*) with different regressors making use of two variations of microstructure representations. The six systems from the *very left bar* to the very right correspond to Ex 2b, 2c, 3a −1, 3a −2, 3b −1, and 3b −2, while the training time depicted is an average of each adjacent pair under the same regressor. The reason for using the average is that the difference of feature sizes under the same model type is relatively trivial

The effect of different regression models, each exploring two feature sets, is demonstrated in Fig. 9, comparing with two corresponding models (that have used 57 and 93 features) from Ex 2. Only test performances are shown in this comparison, and the MASE is the average among the entire 1000 test MVEs. The ensemble model RF gives the best test performance in both feature sets. It is once again observed that including too many features only deteriorates the accuracy. Although as many as 50 regression trees are built in RF, due to the subsampling of data space, the increase in training time compared to a single tree in the case of M5 is only moderate.

**Fig. 10:**
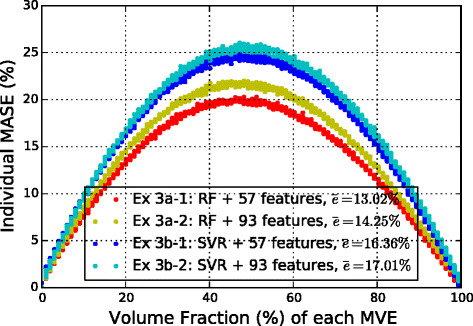
Influence of volume fraction on the error for Ex 3. Individual MASE for each of the 1000 MVEs in test set given by the four systems in Ex 3 are shown

**Fig. 11:**
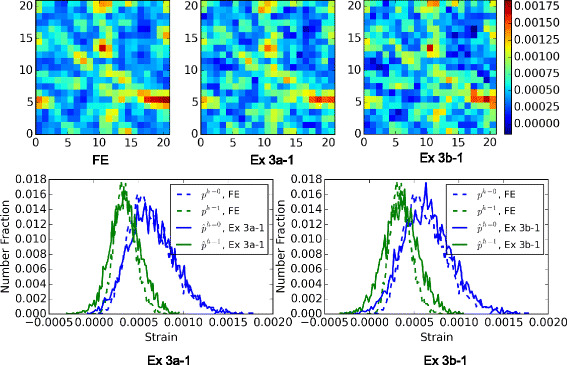
Comparison of strain field predictions. A comparison of FE and statistical model predictions of the strain fields, $\hat {p}$, in an example MVE with the volume fraction of 50 %. The models are those developed in Ex 3a −1 and Ex 3b −1. *Strain fields* on a center slice are compared at the *top*. *Histogram plots* of strain distributions are presented at the *bottom*. One hundred bins are used to generate each distribution curve for each phase

A more detailed comparison of the individual MASE for each of the 1000 MVEs, with respect to volume fractions, is shown in Fig. 10. And Fig. 11 compares the predicted strain fields with FE results for the same MVE and slice as in Fig. 8. Only the two best models, Ex 3a −1 and Ex 3b −1 that both use 57 features, are selected to show. Once again, it is observed that the accuracy of the models in predicting the spatial locations and distributions of the hot spots has improved significantly in these new models compared to the earlier ones.

## Conclusions

In this paper, we explored multiple data mining experiments and strategies for establishing statistical models for capturing elastic localization relationships in high contrast composites. More specifically, our focus was on a composite with a contrast of 10. The efficacy of different approaches for feature selection and regression were studied systematically. We demonstrated that a set comprised of basic feature descriptors combined with engineered (constructed) features is able to boost the prediction performance. Moreover, a reduced set of descriptors generated by feature ranking methods offers even better results. In terms of regression techniques, ensemble methods such as random forests show superiority when both accuracy and time consumption are taken into account.
